# Association between Elevated TNF-α Levels and Severe Malaria

**DOI:** 10.31661/gmj.v12i.2927

**Published:** 2023-04-25

**Authors:** Khalid Abdelsamea Mohamedahmed

**Affiliations:** ^1^ Department of Hematology and Immunohematology, Faculty of Medical Laboratory Sciences, University of Gezira, Wad Medani, Sudan; ^2^ Department of Hematology and Immunology, Faculty of Medical Laboratory Sciences, University of Gezira, Wad Medani, Sudan

**Keywords:** TNF-α, Severe, Malaria, Proinflammatory Cytokine

## Dear Editor,

Tumor necrosis factor-alpha (TNF-α) is predominantly produced by γδ T cells and CD14+ monocytes during immune responses to malaria and helps in the control of parasitemia. TNF-α and interferon-gamma (IFN-γ) act synergistically to optimize nitric oxide (NO) production which in turn leads to parasite killing. Also, TNF-α enhances human neutrophil killing of the plasmodium parasite [1].

TNF-α plays important role in the pathogenesis of multiple inflammatory disorders, autoimmune diseases, and infectious diseases, including malaria [2][3].

At low levels, TNF-α is believed to augment parasite killing by macrophage activation and subsequent release of cytokines, whereas high TNF-α level has been associated with severe manifestations. Individual variation in TNF-α production mainly by macrophages and natural killer (NK) cells is likely to influence severe disease manifestation [2].

Severe malarial infection is associated with the rupture of parasitized red blood cells releasing malaria pigment and other soluble antigens and toxins that stimulate the overproduction of TNF-α in human monocytes and may also stimulate intense T helper type 1-like response locally, in tissues of vital organs which result in upregulation of expression of endothelial adhesion molecules such as intracellular adhesion molecule-1 (ICAM-1) or other adhesion molecules.

This in turn leads to increased and enhanced parasite adherence and sequestration of parasitized red cells that leads to subsequent microvascular obstruction, decreasing oxygen delivery, and/or possibly the release of NO from the endothelium which may ultimately contribute to the pathogenesis [4].

Also, elevated TNF-α levels stimulated phagocytosis and thereby enhancing clearance of parasitized erythrocytes but the prolonged response was associated with severe disease syndromes.

TNF-α has been shown to increase the severity of inflammation by inducing cyclooxygenase-2 (COX-2) and subsequently generating effector molecules, such as prostaglandins. Many of the signs and symptoms and complications associated with malaria to be linked to TNF-α [4][5].

TNF-α is considered to be important for parasite destruction and elimination, as well as in the development of fever and other clinical symptoms, and it also contributes to the development of severe malaria disease [6]. TNF-α could affect the outcome of malaria in several ways.

TNF-α promotes fever, which may suppress parasite growth, and it also induces the expression of adhesion molecules and proinflammatory molecules [7]. Lethal cerebral malaria (CM) has been associated with a high level of TNF-α in serum [7].

The TNF-α overproduction in malaria can contribute to reduced red cell production and anemia through suppression of bone marrow erythropoiesis and dyserythropoiesis [1][8]. The overproduction of TNF-α could be associated with more rapid resolution of fever and parasite clearance but predisposes to severe pathology of disease [9].

Severe malaria is associated with several genes, including TNF-α gene polymorphisms. TNF-α is thought to be a critical factor in malaria pathogenesis, the control of parasitemia, and increased susceptibility to severe malaria [2][10].

High TNF-α plasma levels have been associated with increased susceptibility to severe malaria [2][10]. The variation in the TNF-α gene phenotypes related to malaria infection and severe disease [10].

In conclusion, tumor necrosis factor (TNF-α) is a common proinflammatory cytokine. It plays a central role in malaria pathogenicity either in the cure or complication of malaria. Their high level is associated with the severe outcome of malaria.

## Conflict of Interest

The author has declared that no Conflict of Interest exist.
